# Investigating the Effects of Food Available and Climatic Variables on the Animal Host Density of Hemorrhagic Fever with Renal Syndrome in Changsha, China

**DOI:** 10.1371/journal.pone.0061536

**Published:** 2013-04-24

**Authors:** Hong Xiao, Hai-Ning Liu, Li-Dong Gao, Cun-Rui Huang, Zhou Li, Xiao-Ling Lin, Bi-Yun Chen, Huai-Yu Tian

**Affiliations:** 1 College of Resources and Environment Science, Hunan Normal University, Changsha, China; 2 Hunan Provincial Center for Disease Control and Prevention, Changsha, China; 3 Centre for Environment and Population Health, School of Environment, Griffith University, Brisbane, Queensland, Australia; 4 College of Resources Science and Technology, Beijing Normal University, Beijing, China; 5 Beijing Energy Conservation and Environmental Protection Center, Beijing, China; Tulane School of Public Health and Tropical Medicine, United States of America

## Abstract

**Background:**

The transmission of hemorrhagic fever with renal syndrome (HFRS) is influenced by population dynamics of its main host, rodents. It is therefore important to better understand rodents’ characteristic in epidemic areas.

**Methodology/Principal Findings:**

We examined the potential impact of food available and climatic variability on HFRS rodent host and developed forecasting models. Monthly rodent density of HFRS host and climate data in Changsha from January 2004 to December 2011 were obtained. Monthly normalized difference vegetation index (NDVI) and temperature vegetation dryness index (TVDI) for rice paddies were extracted from MODIS data. Cross-correlation analysis were carried out to explore correlation between climatic variables and food available with monthly rodent data. We used auto-regressive integrated moving average model with explanatory variables to examine the independent contribution of climatic variables and food supply to rodent density. The results indicated that relative rodent density of HFRS host was significantly correlated with monthly mean temperatures, monthly accumulative precipitation, TVDI and NDVI with lags of 1–6 months.

**Conclusions/Significance:**

Food available plays a significant role in population fluctuations of HFRS host in Changsha. The model developed in this study has implications for HFRS control and prevention.

## Introduction

Hemorrhagic fever with renal syndrome (HFRS), a rodent-borne viral disease caused by different species of hantaviruses, is characterized with fever, hemorrhage, headache, back pain, abdominal pain, and acute kidney injury [1]. HFRS, initially described clinically at the turn of the 20th century,is primarily distributed in the Asian and European continents [2]. In western and central Europe one of the most important hantavirus is Puumala virus [3], [4], while in China there are two predominant species of hantavirus, Hantaan and Seoul virus, each of which has co-evolved with a distinct rodent host [5].

HFRS is a serious disease in China, at present, it is endemic in all 31 provinces, autonomous regions and metropolitan areas in mainland China where human cases account for 90% of the total global cases [6]. Through a series of measures that improve environment, vaccinate and control population of rodents, there is a trend towards declined incidence of HFRS in China, but it is still the highest incidence of HFRS in the world, and a total of 53,471 cases were reported from 2006 to 2010 in China [7]. Hunan Province is the province with one of the highest incidence of HFRS in China, and its capital city Changsha bears a large HFRS burden in Hunan Province. The highest incidences in Ningxiang county within Changsha was recorded as 101.68 per 100,000 in 1994 [8].

HFRS is transmitted to human by contact with rodent urine, feces or saliva [3], [9]. For this reason, the fluctuations in abundance of rodent host are considered as an important reason for temporal variations in human infections of HFRS. Human nephropathia epidemica (NE) epidemics, a mild form of HFRS, have been observed a close relation with bank vole populations in many countries in Europe [10], [11], [12]. Human hantavirus epidemics can be accurately predicted solely by the population dynamics of the host, even without knowledge of the degree of hantavirus infection of the involved rodent reservoir [13]. In recent years, the relationship between HFRS and host population have been observed in China as well. The rise and falls of incidence rate was found generally coincident with rodent density, and a statistical correlation was presented between the incidence rate and rodent density [14]. It is therefore important to know more about the laws between rodents and environment, which have implications for HFRS control and prevention. Population fluctuations generally are driven by a combination of multiple environmental factors [15], [16]. The climatic variables and food supply are important indicator for the rodent population [17]. the close relationship among rodent population, rainfall and food was found by compared the two rodent species utilizing exploratory analyses of species densities with time series statistical tools [18]. It is therefore necessary to have more knowledge about the dynamics of the rodent host and their interactions with natural environment. However, the quantitative relationship among climatic variables, food available and host population remains to be determined.

The aim of this study is to investigate the effects of climatic variables and food available on the host density of hemorrhagic fever with renal syndrome using data from rodent host population dynamics, climatic variables and food available in Changsha. Firstly, time series analyses of monthly rodent data with climatic variables and food available were carried out using autocorrelation analysis and cross-correlation analysis. Secondly, built up Auto Regressive Integrated Moving Average (ARIMA) model to examine the independent contribution of climatic variables and food available to HFRS host dynamics. Finally, we forecasted the changed trend of the rodent population.

## Materials and Methods

### Study Area

**Figure 1:**
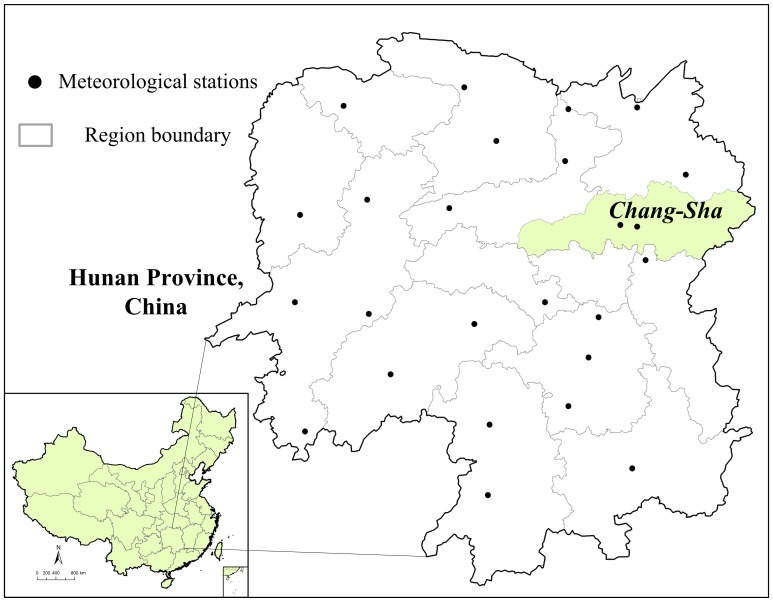
The study area, Changsha, China.

The study area covers Changsha, the capital city of Hunan Province in Central China, located between latitude 27°51′ and 28°40′ north, and longitude 114°15′ and 111°53 (Figure 1). Changsha has a humid subtropical climate, with annual average temperature being 17.2°C. Average annual precipitation is 1, 390 mm.

### Rodent Data

We conducted a density-of-rodents survey in the residential areas, industrial areas and fields where the rodents may haunt. Rodents of hantaviruses were trapped in Changsha every month from January 2004 to December 2011. There were 19 permanent trapping sites, and a total of 50, 376 traps were set from 2004 to 2011. At least 200 traps were placed at each trapping site each night and received in the morning, which is conducted over three consecutive nights: one trap every 5 meters in each row with 50 meters between rows. We used the “Relative rodent density” to describe the combined effect of rodent density:
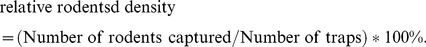
(1)


The number of rodents captured divided by the number of traps placed is that month’s density of rodents. The species of rodent was also identified and Shannon-Wiener diversity index was used to calculate species diversity:
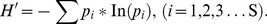
(2)Where where *p* is proportion of the total number of individuals belonging to species *i* and S is is species richness.

### Meteorological Data

From 2004 to 2011, monthly climate data in Changsha were collected from the China Meteorological Data Sharing Service System (http://cdc.cma.gov.cn/index.jsp). The climate variables included monthly mean temperature (MT), monthly mean maximum temperature (MaxT), monthly mean minimum temperature (MinT), and monthly accumulative precipitation (AP).

### Land-surface Attributes for Food Available

**Figure 2:**
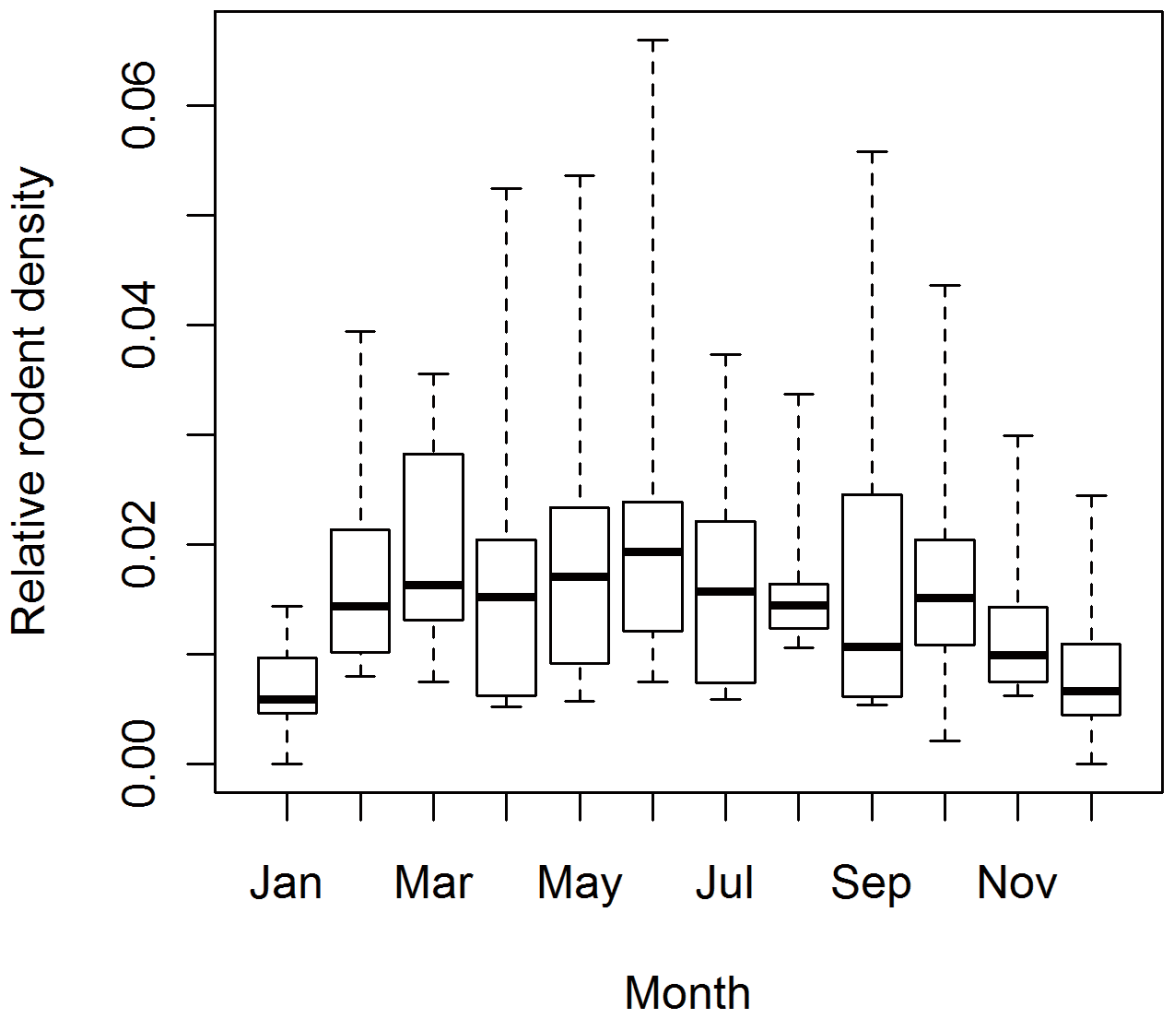
Annual trend of the relative density of rodent host of HFRS in Changsha, 2004–2011.

Two indices that characterize habitat quality were extracted from monthly MODIS (Moderate-Resolution Imaging Spectroradiometer) data from January 2004 to December 2011 (Figure 2). The MODIS data (MOD11A2 and MOD13A2) was acquired with a spatial resolution of 1000-m from the International Scientific Data Service Platform (http://datamirror.csdb.cn). In this study, MODIS data for the study area were transformed to the UTM-WGS84 50N projection. The land use data are from the Second National Land Survey Data. NDVI and TVDI value for rice paddies were used to reflect the lushness of the vegetation, thus these indices are good indicators of the food available for rodent.

The normalized difference vegetation index (NDVI) was referred to as a greenness index which represents the vegetation amount and reflects agricultural biomass. The NDVI is calculated using the near-infrared (NIR) and red reflectance bands:
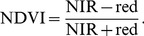
(3)Where red and NIR stand for the spectral reflectance measurements acquired in the red and near-infrared regions, respectively.

The temperature vegetation dryness index (TVDI) has been widely used in soil moisture estimation [19]. The TVDI is estimated using the following equation:
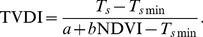
(4)Where Ts is the observed LST at a given pixel, Ts_min_ is the minimum land surface temperature (LST) for a given NDVI, defining the wet edge, a and b are parameters defining the dry edge, modeled as a linear fit to data (Ts_max_ = a+bNDVI). The TVDI is higher for dry and lower for wet conditions and varies between 0 and 1.

### Data Analysis

Time series analyses of monthly rodent data with climatic variables and food available were carried out using autocorrelation analysis and cross-correlation analysis to examine the seasonal and lagged effects in the data sets. The cross-correlation analysis was performed as follows: first, convert one of the series into white noise, and then the second series was filtered by the same filter before computation. The significance of the cross-correlations was assessed on the basis of its two standard error limits (significant at 0.05 level). Climatic variables that did not exhibit significant cross-correlations with the rodent data were excluded from further analysis.

In this study, which incorporates climatic input series is referred as ARIMAX [20], was used to examine the independent contribution of climatic variables and food available to HFRS host dynamics. SARIMA is the ARIMA model that incorporates seasonality, referred as SARIMA(p, d, q)(P, D, Q), where p indicates the AR order, d the differencing order and q the MA order. P, D and Q indicate the seasonal order of AR, differencing, and MA, respectively. Autocorrelation function (ACF) and partial autocorrelation function (PACF) were performed to analyze any random, stationary and seasonal effects on the time series data. The residuals were further inspected for autocorrelation through ACF and PACF. Goodness of fit was examined through calculated Akaike’s information criterion (AIC) and the mean relative prediction error (MRPE).
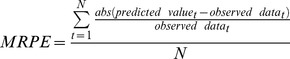
(5)


The monthly rodents survey data was divided into two sets: one was used in the fitting process (parameter estimation), and another for prediction. We took the observations in the latest one year as the prediction period. Among the 96 observations in Changsha survey data, we used 84 points for fitting and 12 for prediction. All ARIMA modeling were performed using SAS software, Version 9.1.3 of the SAS System for Windows (SAS Institute, Inc., Cary, NC).

## Result

### Description of Population Dynamics

**Figure 3:**
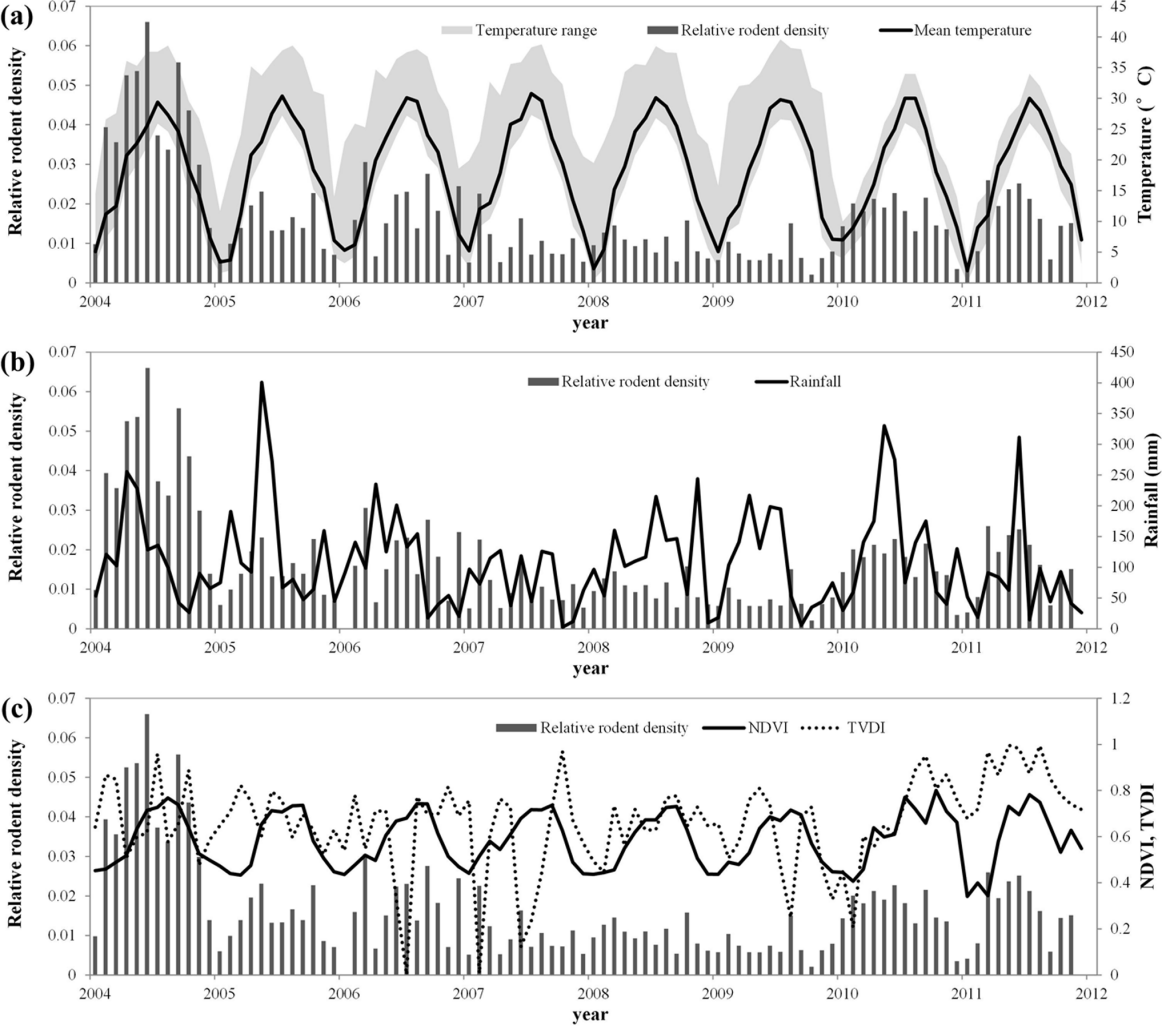
Environmental variables in Changsha. **(a) monthly average of temperature, (b) monthly accumulated rainfall and (c) monthly NVDI and TVDI for rice paddies.**

A total of 812 rodents were captured in residential areas industrial areas and fields over the study period. The monitoring data shows *Rattus norvegicus* (55.58%) and *Mus musculus* (26.72%) were the most predominant species captured which are the predominant virus host species. Shannon-Wiener diversity index and evenness index of rodent in Changsha from 2004 to 2011 were 0.86 and 0.78 respectively. The highest species diversity of 1.06 was found in 2011. Annual averages across years reveal that major peak months over the study period occurred in June, September and October (Figure 2, Figure 3).

### Climate and Food Available with Population Fluctuations

As shown in Table 1, there was a positive correlation between monthly mean temperatures and monthly accumulative precipitation with population density of HFRS host, with the highest correlation coefficients having a lag of 5 and 1 months, respectively. NDVI was positively associated with density of HFRS host with a lag of 3 months. However, there was an inverse correlation between TVDI and rodent density.

**Table 1:** Cross-correlations between pre-whitened series and the rodent density.

Variable	Lag					
	0	1	2	3	5	6
MT	0.233*	0.147	0.167	−0.215*	−0.033	0.299**
AP	0.05	0.214*	0.042	0.087	−0.119	−0.173
NDVI	0.157	0.177	0.072	0.214*	−0.078	−0.036
TVDI	−0.189	0.168	0.009	0.082	−0.012	0.051

* *p*<0.05,

** *p*<0.01.

Based on the ACF and PACF, we fitted several univariate (S)ARIMA models and found that the best performing models are ARIMA(1,1,2). Thus we chose ARIMA(1,1,2) as the baseline model because all *p*-values of the estimated coefficient are relatively significant (*p*<0.05) (Table 2).

**Table 2:** Summary of model performance and the estimated coefficients.

Model	Fit		Prediction	AR		MA		Environmental variables		
	MPRE	AIC	MPRE	Est.	Pr>|t|	Est.	Pr>|t|	Vars	MPRE	AIC
ARIMA(1,1,2)	0.2252	−624.462	0.1861	0.8237	<.0001	1.2375	0.9606			
						−0.2375	0.9683			
SARIMA(1,1,2)(0,1,1)	0.7375	−531.519	0.4723	−0.5405	<.0001	0.2558	0.0289			
						0.5937	<.0001			
ARIMA(1,1,2) with NDVI	0.2821	−606.063	0.3139	0.8843	<.0001	1.2747	0.9666	NDVI(Lag3)	0.0225	0.0268
						−0.2748	0.9738			
SARIMA(1,1,2)(0,1,1) with NDVI	0.7308	−530.312	0.4262	−0.5816	<.0001	0.28207	0.0205	NDVI(Lag1)	0.04091	0.0064
						0.5177	<.0001			
ARIMA(1,1,2) with AP	0.2801	−620.177	0.2233	0.8289	<.0001	1.2807	0.0007	AP(Lag1)	1.87E-05	0.0903
						−0.2807	<.0001			
SARIMA(1,1,2)(0,1,1) with AP	0.7415	−508.31	0.4937	−0.5267	<.0001	0.22329	0.0674	AP(Lag3)	−5.31E-07	0.9638
						0.61085	<.0001			
ARIMA(1,1,2) with LST	0.2499	−584.528	0.4730	−0.8302	0.0938	−0.3908	0.4195	LST(Lag5)	−0.000109	0.4562
						0.4143	0.0398			
SARIMA(1,1,2)(0,1,1) with LST	0.7425	−523.251	0.4939	−0.5344	<.0001	0.23855	0.041	LST(Lag1)	0.000227	0.6549
						0.61492	<.0001			
ARIMA(1,1,2) with TVDI	0.1969	−617.186	0.1605	0.8956	<.0001	1.2261	0.0068	TVDI(Lag1)	0.006809	0.1313
						−0.2261	<.0001			
SARIMA(1,1,2)(0,1,1) with TVDI	0.7502	−509.659	0.5191	−0.5246	<.0001	0.24943	0.0395	TVDI(Lag3)	0.005443	0.2321
						0.62236	<.0001			
ARIMA(1,1,2) with NDVI and AP	0.3002	−605.724	0.3291	0.8759	<.0001	1.257	0.9536	NDVI(Lag3)	0.01937	0.0673
						−0.2571	0.9632	AP(Lag1)	0.000014	0.2214
SARIMA(1,1,2)(0,1,1) with NDVI and AP	0.7354	−513.207	0.3031	−0.5852	<.0001	0.27536	0.0292	NDVI(Lag1)	0.04116	0.008
						0.5162	<.0001	AP(Lag3)	−2.83E-06	0.8049
ARIMA(1,1,2) with NDVI and LST	0.3015	−592.734	0.3752	0.8629	<.0001	1.358	0.9317	NDVI(Lag3)	0.02471	0.0124
						−0.3581	0.9499	LST(Lag5)	−0.0001796	0.2322
SARIMA(1,1,2)(0,1,1) with NDVI and LST	0.7309	−528.319	0.4294	−0.5810	<.0001	0.27931	0.0227	NDVI(Lag1)	0.04072	0.0073
						0.51867	<.0001	LST(Lag1)	0.0000399	0.936
ARIMA(1,1,2) with NDVI and TVDI	0.2610	−607.672	0.3490	0.8461	<.0001	1.2057	0.991	NDVI(Lag3)	0.02432	0.0189
						−0.2057	0.9926	TVDI(Lag1)	0.00784	0.0788
SARIMA(1,1,2)(0,1,1) with NDVI and TVDI	0.7505	−514.264	0.6376	−0.5729	<.0001	0.29805	0.0167	NDVI(Lag1)	0.04031	0.0093
						0.52946	<.0001	TVDI(Lag3)	0.0047314	0.2844
ARIMA(1,1,2) with AP and LST	0.3016	−586.775	0.5045	−0.8169	0.0351	−0.3636	0.3329	AP(Lag1)	0.0000233	0.0427
						0.4387	0.0079	LST(lag5)	−0.0001658	0.2357
SARIMA(1,1,2)(0,1,1) with AP and LST	0.7465	−506.549	0.4962	−0.5239	<.0001	0.20612	0.0918	AP(Lag3)	−1.32E-06	0.911
						0.62248	<.0001	LST(Lag1)	0.0002675	0.6155
ARIMA(1,1,2) with AP and TVDI	0.3419	−620.811	0.3536	0.7796	<.0001	1.1943	0.9818	AP(Lag1)	0.0000182	0.0958
						−0.1943	0.9848	TVDI(Lag1)	0.0076632	0.0926
SARIMA(1,1,2)(0,1,1) with AP and TVDI	0.7503	−507.66	0.5192	−0.5250	<.0001	0.24937	0.041	AP(Lag3)	−2.60E-07	0.9824
						0.62256	<.0001	TVDI(Lag3)	0.0054414	0.2354
ARIMA(1,1,2) with LST and TVDI	0.2285	−583.938	0.4984	−0.7560	0.0919	−0.36078	0.4084	LST(Lag5)	−0.0000856	0.5677
						0.38558	0.0216	TVDI(Lag1)	0.005924	0.2153
SARIMA(1,1,2)(0,1,1) with LST and TVDI	0.7513	−507.87	0.5199	−0.5209	<.0001	0.23543	0.0522	LST(Lag1)	0.0002476	0.6398
						0.6335	<.0001	TVDI(Lag3)	0.0054056	0.2365
ARIMA(1,1,2) with NDVI and AP and LST	0.3612	−592.425	0.4661	0.8566	<.0001	1.37254	0.9276	NDVI(Lag3)	0.02147	0.0361
						−0.37262	0.9475	AP(Lag1)	0.0000144	0.22
								LST(Lag5)	−0.0002122	0.1526
SARIMA(1,1,2)(0,1,1) with NDVI and AP and LST	0.7365	−511.242	0.3066	−0.5840	<.0001	0.26806	0.0349	NDVI(Lag1)	0.04075	0.0091
						0.51939	<.0001	AP(Lag3)	−3.12E-06	0.7874
								LST(Lag1)	0.0000985	0.8502
ARIMA(1,1,2) with NDVI and AP and TVDI	0.2926	−608.511	0.3920	0.7496	<.0001	1.1503	0.9409	NDVI(Lag3)	0.02174	0.0447
						−0.1504	0.9495	AP(Lag1)	0.0000132	0.2345
								TVDI(Lag1)	0.0087602	0.0527
SARIMA(1,1,2)(0,1,1) with NDVI and AP and TVDI	0.7373	−512.305	0.3237	−0.5757	<.0001	0.29618	0.0184	NDVI(Lag1)	0.04049	0.0095
						0.52923	<.0001	AP(Lag3)	−2.31E-06	0.8411
								TVDI(Lag3)	0.0046925	0.2911
ARIMA(1,1,2) with NDVI and LST and TVDI	0.3054	−593.062	0.4049	0.7824	<.0001	1.2536	0.9555	NDVI(Lag3)	0.02666	0.0105
						−0.25367	0.9647	LST(Lag5)	−0.0001626	0.312
								TVDI(Lag1)	0.0076007	0.1021
SARIMA(1,1,2)(0,1,1) with NDVI and LST and TVDI	0.7355	−512.28	0.3270	−0.5720	<.0001	0.29374	0.019	NDVI(Lag1)	0.04003	0.0105
						0.53184	<.0001	LST(Lag1)	0.0000646	0.9009
								TVDI(Lag3)	0.0047176	0.2887
ARIMA(1,1,2) with AP and LST and TVDI	0.2851	−586.275	0.5240	−0.7553	0.0315	−0.3492	0.3028	AP(Lag1)	0.0000232	0.0419
						0.4147	0.0032	LST(Lag5)	−0.0001415	0.3257
								TVDI(Lag1)	0.0059708	0.2038
SARIMA(1,1,2)(0,1,1) with AP and LST and TVDI	0.7529	−505.876	0.5201	−0.5221	<.0001	0.23491	0.0546	AP(Lag3)	−9.66E-07	0.9351
						0.63445	<.0001	LST(Lag1)	0.000253	0.6364
								TVDI(Lag3)	0.0053979	0.2403
ARIMA(1,1,2) with NDVI and AP and LST and TVDI	0.3126	−592.727	0.4159	0.7832	<.0001	1.2408	0.9506	NDVI(Lag3)	0.02309	0.0331
						−0.2409	0.9604	AP(Lag1)	0.0000141	0.2213
								LST(Lag5)	−0.0001753	0.2811
								TVDI(Lag1)	0.0075494	0.1024
SARIMA(1,1,2)(0,1,1) with NDVI and AP and LST and TVDI	0.7369	−510.329	0.3261	−0.5748	<.0001	0.2907	0.0215	NDVI(Lag1)	0.04016	0.0108
						0.5322	<.0001	AP(Lag3)	−2.54E-06	0.8274
								LST(Lag1)	0.0000799	0.8789
								TVDI(Lag3)	0.0047	0.2962

Abbreviations: ARIMA = Autoregressive Integrated Moving Average; S = Seasonal; X = with explanatory variables; LST = Land Surface Temperature; AP = Accumulative Precipitation; NDVI = Normalized Difference Vegetation Index; TVDI = Temperature Vegetation Dryness Index; MRPE = Mean Relative Prediction Error; AIC = Akaike’s Information Criterion; AR = Autoregressive coefficients; MA = Moving Average Coefficients; Est = Estimated values through conditional least square method.

We further fitted ARIMAX model with the lagged climate and land-surface variables as input series, and the results are summarized in Table 2. For these multivariate models, the best fit MRPE is obtained from ARIMAX(1,1,2) with TVDI as covariate and ARIMAX(1,1,2) model has the best AIC. Comparing the ARIMAX(1,1,2) with TVDI (lag-3) model with the baseline univariate model discussed previously (ARIMA(1,1,2)), we found that including the TVDI improve the fit RMSE by 14.36% and the prediction RMSE by 15.89% from the baseline model.

**Figure 4:**
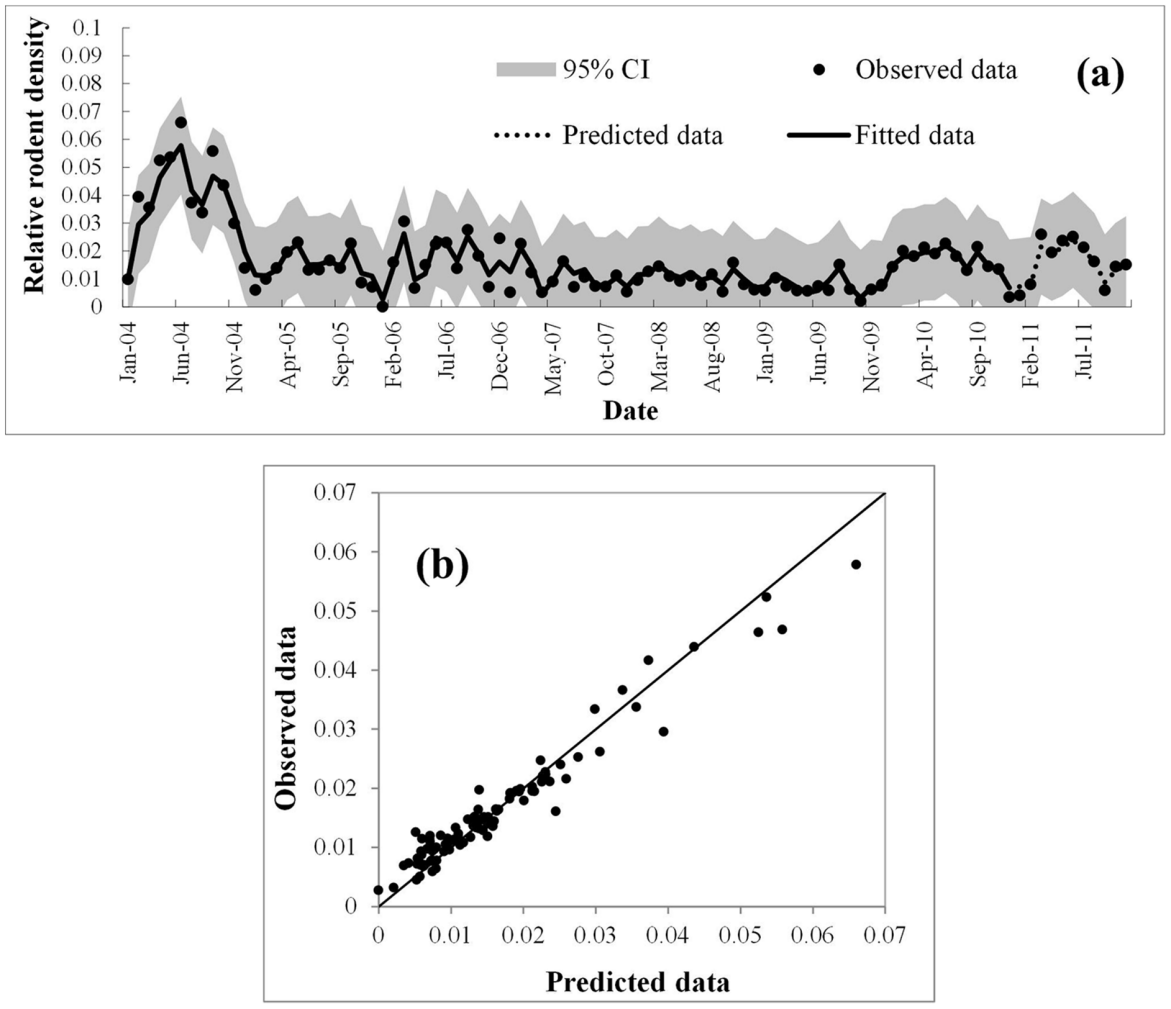
Observed versus predicted rodent density in Changsha. **(a) Temporal dynamics and (b) scatterplot.**

We choose the ARIMAX(1,1,2) with TVDI because it has the lower fit RMSE. The biological meanings of the model is that the the current density of rodents depends on both past density of rodents and past three months of TVDI value. The fitted and predicted values of this model were plotted in Figure 4. The observed and predicted number of population density from the final model matched reasonably well for Changsha, as did the 1-year forecast. The MRPE of the model was 16.05% and the goodness-of-fit analyses showed no significant autocorrelation between residuals at different lags in the final model.

## Discussion

Through the use of ARIMA models, we first examined whether rodent density can be modeled as a univariate (S)ARIMA. The results indicated that the ARIMA was capable of forecasting 1-step ahead future rodent density relatively well. The best univariate model is ARIMA(1,1,2), where rodent density depends on density in previous one month. In the ARIMAX models, we found that TVDI for rice paddies is a significant predictor for rodent density in Changsha. A key finding from this study was that food supply is an important predictor of the rodent population dynamics of the host in central China. The results clearly demonstrated that host density of HFRS was well predicted by the climatic factors and food supply conditions and we found a consistent relationship between these factors with lags of 1–6 months and rodent density. The main natural reservoir of hantaviruses is rodent, human can be infected directly or indirectly through contact with rodents [21], thus human HFRS cases are associated with the population of reservoir hosts [22]. Reservoir hosts are the potential indicator of hantavirus emergence [23], increased rodent density increases the probability of human contact with rodents, and we can predict potential HFRS incident by monitoring rodent density. So this lead time is of particular importance in predicting the possible surge in host population and the following epidemics of HFRS.

The significant variables in the final ARIMAX model could offer a strong explanation for dynamics changes of HFRS host population. Climate variables were excluded from the final ARIMAX model, although climate series were significantly correlated with the host population. The question is then to identify the driving factors behind these fluctuations of rodent density, most rodent species responded directly to fluctuations in food available [17], [18], the densities of these rodent hosts were driven by changes in food resources [24]. The fluctuations of food availability can be linked to environmental influences, some of which can be related to climate change. Previous study found that food availability was closely associated with Southern Oscillation Index and NE winds [25]. The food availability depended on the local climate, and then can decide carrying capacity in an area [26]. Under optimal weather conditions, higher carrying capacity can afford bigger population size of rodent. The results indicated that weather may affect the rodent reservoir indirectly through its effect on the food available condition.

Temperature vegetation dryness index is a complex variable, which reflect the moisture condition, temperature, lushness of the vegetation. We thought it was a good indicator of food available for the rodent, whereas it was directly influenced by climatic forcing [16]. The TVDI was negatively associated with the density of HFRS host, because higher TVDI value represents dry conditions lead to the low biomass and food shortage. The population of rodents thus decrease. When the opposite happens, rodent population increase. The results of the current study are helpful in defining significant exogenous factors on the population dynamics of HFRS host. ARIMAX model with TVDI may provide an expert tool to predict the population fluctuations of HFRS host by making use of remote sensing tools and climatological data. However, Changsha is located in the humid subtropical climate area, the laws between rodents and TVDI need to be further investigated in other areas.

The limitations of this study should also be acknowledged. In this study, we only analyzed the role of exogenous factors in population dynamics, without the endogenous factors (e.g., competition, predation) [15]. Because both types of factors influence population dynamics [27]. In future research, we might try to analyze some exogenous factors in the model, and find the relationships between rodent population and TVDI in other larger areas.

In conclusion, this study suggest that antecedent patterns of food supply were the key determinants of the HFRS host population in Changsha, China. The forecasting model of this study provides an predictive capacity for potential HFRS epidemics, which can give health authorities sufficient time to formulate plans, disseminate warnings, and implement public health interventions. There is also an urgent need for monitoring and predicting HFRS incidence to reduce the substantial disease burden caused by HFRS.
