# Correction: Investigating the Effects of Food Available and Climatic Variables on the Animal Host Density of Hemorrhagic Fever with Renal Syndrome in Changsha, China

**DOI:** 10.1371/annotation/524770c1-d027-4543-92a5-ede5270adeef

**Published:** 2013-09-09

**Authors:** Hong Xiao, Hai-Ning Liu, Li-Dong Gao, Cun-Rui Huang, Zhou Li, Xiao-Ling Lin, Bi-Yun Chen, Huai-Yu Tian

Two funding organizations were incorrectly omitted from the Funding Statement. The Funding Statement should read: "This work was supported by the Key Discipline Construction Project in Hunan Province (2008001), Scientific Research Fund of the Hunan Provincial Education Department (11K037), Hunan Provincial Natural Science Foundation of China (11JJ3119), Science and Technology Planning Project of Hunan Province, China (2010SK3007), Key Subject Construction Project of Hunan Normal University (geographic information systems), Key Project of Hunan Provincial Education Department (13A051) and National Natural Science Foundation of China. The funders had no role in study design, data collection and analysis, decision to publish, or preparation of the manuscript." 

